# Validation of a German translation of the CARE questionnaire and its implementation as electronic PROM to assess patient-reported postoperative convalescence and recovery after major urological surgery

**DOI:** 10.1007/s00345-021-03713-6

**Published:** 2021-05-08

**Authors:** Frederik Wessels, Maximilian Lenhart, Manuel Neuberger, Julia Mühlbauer, Johannes Huber, Johannes Breyer, Philipp Nuhn, Maurice S. Michel, Julian Koenig, Maximilian C. Kriegmair

**Affiliations:** 1 Department of Urology and Urological Surgery, Medical Faculty, University Medical Center Mannheim, Heidelberg University, Theodor-Kutzer-Ufer 1-3, 68167 Mannheim, Germany; 2 Department of Urology, University Hospital Carl Gustav Carus Dresden, TU Dresden, Dresden, Germany; 3 Department of Urology, University of Regensburg, Caritas St. Josef Medical Center, Regensburg, Germany; 4 University Hospital of Child and Adolescent Psychiatry and Psychotherapy, University of Bern, Bern, Switzerland; 5 Section for Experimental Child and Adolescent Psychiatry, Department of Child and Adolescent Psychiatry, Centre for Psychosocial Medicine, University of Heidelberg, Heidelberg, Germany

**Keywords:** Prostatectomy, Nephrectomy, Cystectomy, Patient-reported outcome, Outcome measures, Quality of life, CARE

## Abstract

**Purpose:**

To validate a German translation of the convalescence and recovery evaluation (CARE) as an electronic patient-reported outcome measure (ePROM) and use it to assess recovery after major urological surgery.

**Methods:**

The CARE questionnaire was provided to patients scheduled for major urological surgery preoperatively, at discharge and 6 weeks postoperatively, using an ePROM system. Cronbach’s alpha, inter-scale correlations and confirmatory factor analysis (CFA) were used to validate the translation. Mixed linear regression models were used to identify factors influencing CARE results, and a multivariable logistic regression analysis was done to determine the predictive value of CARE results on quality of life (QoL).

**Results:**

A total of 283 patients undergoing prostatectomy (*n* = 146, 51%), partial/radical nephrectomy (*n* = 70, 25%) or cystectomy (*n* = 67, 24%) responded to the survey. Internal consistency was high (*α* = 0.649–0.920) and the CFA showed a factor loading > 0.5 in 17/27 items. Significant main effects were found for the time of survey and type of surgery, while a time by type interaction was only found for the gastrointestinal subscale ($\chi_{(4)}^{2}$ = 30.37, *p* < *0.0001*) and the total CARE score (TCS) ($\chi_{(4)}^{2}$ = 13.47, *p* = *0.009*) for cystectomy patients, meaning a greater score decrease at discharge and lower level of recovery at follow-up. Complications demonstrated a significant negative effect on the TCS ($\chi_{(2)}^{2}$ = 8.61, *p* = *0.014*). A high TCS at discharge was an independent predictor of a high QLQ-C30 QoL score at follow-up (OR = 5.26, 95%-CI 1.42–19.37, *p* = *0.013*).

**Conclusion:**

This German translation of the CARE can serve as a valid ePROM to measure recovery and predict QoL after major urological surgery.

## Introduction

The curative treatment of cancerous urological diseases relies on major surgery, such as radical prostatectomy (RP), radical cystectomy (RC) and partial/radical nephrectomy (R/PN). A continuous assessment of traditional clinician-reported outcome measures, such as mortality and complication rates, is required for the evaluation of the quality and success of such operations [1, 2]. Nevertheless, patient-reported outcome measures (PROMs) have been increasingly collected in recent years [3, 4]. The PROMs are standardized and validated tools or questionnaires [5] to assess patient-reported outcomes, such as postoperative quality of life (QoL), postoperative health status and others [6]. Several studies have proven the benefit of postoperative patient care when PROMs are routinely used, as their application can lead to better symptom control, an increase in supportive care or a reduction in emergency visits [7, 8].

In urology, several studies have investigated general or cancer-specific QoL after major surgery using PROMs [9, 10]. In contrast, patient-reported postoperative short-term convalescence has been less frequently investigated. Still, to optimize outpatient care after discharge, detailed and current knowledge of the status of recovery is necessary to reduce complications and readmissions [11]. In addition, the application of electronic PROMs (ePROMs) has significantly reduced the barriers to PROM use for both patients and physicians alike. Thus, the ease of implementing PROMs into clinical practice opens up new possibilities in outpatient care. The convalescence and recovery evaluation (CARE) [12] is a multi-dimensional questionnaire that is available to measure convalescence and health status (HS) after surgery, especially after abdominal and pelvic surgery independent of the underlying condition [13]. Studies using CARE after urological surgery were usually designed to compare different surgical techniques [14–16]. Information on the status of recovery at the time of discharge and the further course of recovery is lacking.

Therefore, we aimed to validate a German translation of the CARE questionnaire as an ePROM and to use it to assess self-reported HS after RP, RC and R/PN at the time of discharge and follow-up at a large German university hospital.

## Material and methods

### Study design

Adult patients at the Department of Urology at a German University hospital were prospectively screened for inclusion in this study. For inclusion, patients had to undergo RC, RP or PN/RN (including simple nephrectomy), speak German, have access to the internet, have an email address and be older than 18 years. Patients undergoing emergency surgery and patients with dementia or other cognitive impairment that would prevent the completion of the questionnaires were excluded. This study was approved by the ethical committee (ethic committee II Mannheim, 2018-585N-MA). Patients provided informed consent prior to inclusion.

### Surveys and data collection

In 2019, we introduced a paperless ePROM system at our institution, designed to host ePROMs and the automatically calculated results online (“heartbeat ONE”^1^). Patients willing to participate received a set of questionnaires compiled for the specific operations, including the CARE questionnaire. The patients were able to access the survey via a link sent by email; in addition, on-site completion of the survey was possible using tablets provided by our institution. Our staff provided a short introduction to the easy-to-use system. Additionally, every survey started with a short text explaining the procedure. An exemplary image of the system is shown in Fig. 4 in the Appendix.

At first, only patients undergoing RC and R/PN were included, but in January 2020 patients undergoing RP were additionally enrolled. Patients were required to complete CARE preoperatively (baseline), postoperatively at discharge and 6 weeks after surgery. At least one questionnaire had to be completed for inclusion in the analyses. For patients undergoing RC and R/PN, results on health-related quality of life (HRQoL) 8 weeks after surgery, as measured by the EORTC QLQ-C30 questionnaire [17] on the ePROM system, were also available (*n* = 81).

After a review of the literature, factors that could possibly influence recovery were selected according to the experience of the authors and included age (*n* = 283), comorbidities (measured with the age-adjusted Charlson Comorbidity Index, *n* = 283), sex (*n* = 283), in-hospital complications (*n* = 283) and BMI (*n* = 279). This data was obtained from the medical records.

### The convalescence and recovery evaluation (CARE)

CARE is a validated, multi-dimensional, patient-reported questionnaire in English, first introduced in 2008 and used to assess the HS after surgery [12]. The questionnaire comprises a total of 27 items, each rated on a Likert-type scale. The responses are standardized on a 0–100 scale according to the official scoring instructions [12]. Four subscales can be computed for the HS domains, namely pain (9 items); gastrointestinal (10 items); cognition (4 items); and activity (4 items). The total CARE score (TCS) is derived from the average of all subscales. A higher TCS and higher scores on the respective subscales of CARE indicate a better HS. Since only German-speaking patients were included in this study, author FW translated the questionnaire into German. To evaluate and optimize the precision of the German translation, the questionnaire was translated back into English by author ML and a non-medical bilingual translator. Subsequently, their translation was compared to the original questionnaire. After a review by the involved parties and with a few minor changes, a precise translation could be established. The translation can be found in the supplementary.

### Statistical analysis

JMP 15.2.1 (SAS Institute Inc. 2019, NC, USA) and Stata 16 (StataCorp. 2019. College Station, TX, USA: StataCorp LLC.) were used for the statistical analysis. Mean and standard deviation or median and interquartile range were used to describe the continuous variables. To validate our translated version of CARE, we conducted a confirmatory factor analyses (CFA) using generalized structural equation modelling. Item-loadings were illustrated on the respective subscales of the questionnaire based on all available data (all time-points) using standardized values (maximum-likelihood estimation). Furthermore, we report on global fit indices using the comparative fit index (CFI) and model error using the root mean square error of approximation (RMSEA). In line with existing recommendations [18], we assumed a CFI > 0.90 and a RMSEA below < 0.08 as indicators for good model fit. In line with the initial reporting from Hollenbeck et al., we assessed the inter correlations between the CARE composite (total) and domain (subscales) scores using Pearson correlations separately for all time points. Analyses were based on the (1) available data (completer) and (2) intention-to-treat (ITT) principle. For the ITT analyses, we used the last observation carried forward (LOCF) method to impute missing data. First, mixed-linear regression analyses were used to investigate the main effects of the fixed effects time (baseline [in], discharge [out] and follow-up [FUp]) and type of surgery (nephrectomy, prostatectomy, cystectomy), as well as their interaction on all the CARE subscales and the TCS. The patients’ IDs were used as random factors in all models. Post-hoc pairwise comparisons with Šidák corrected contrasts were used in subsequent analyses. The respective analyses were repeated for both the completer and ITT samples. Second, in exploratory analyses, focusing on the TCS and completer data only, mixed-linear regression analyses were used to investigate each main effect of the fixed effects sex (male, female), age (continuous in years), Boday Mass index (BMI, continuous in kg/m^2^), complication (yes, no) and comorbidity (continuous age-adjusted Charlson Comorbidity Index [19]), as well as their respective interaction with the time of survey. Models were again nested by the patients’ IDs. In exploratory analyses, we did not account for the effect of the type of surgery to avoid overfitting of models but solely focused on main and interaction effects with time. For the visualization of effects from continuous variables (age, BMI, comorbidity), discrete groups were formed, namely age 30–49 years; 50–69 years; 70 + years; BMI: < 25; 25–29; 30+ and comorbidity low (< 3), high (≥ 3). Pearson product moment correlation and multivariable logistic regression analysis were used to investigate the predictive value of HS at discharge on QLQ-C30 outcomes at follow-up.

## Results

**Fig. 1:**
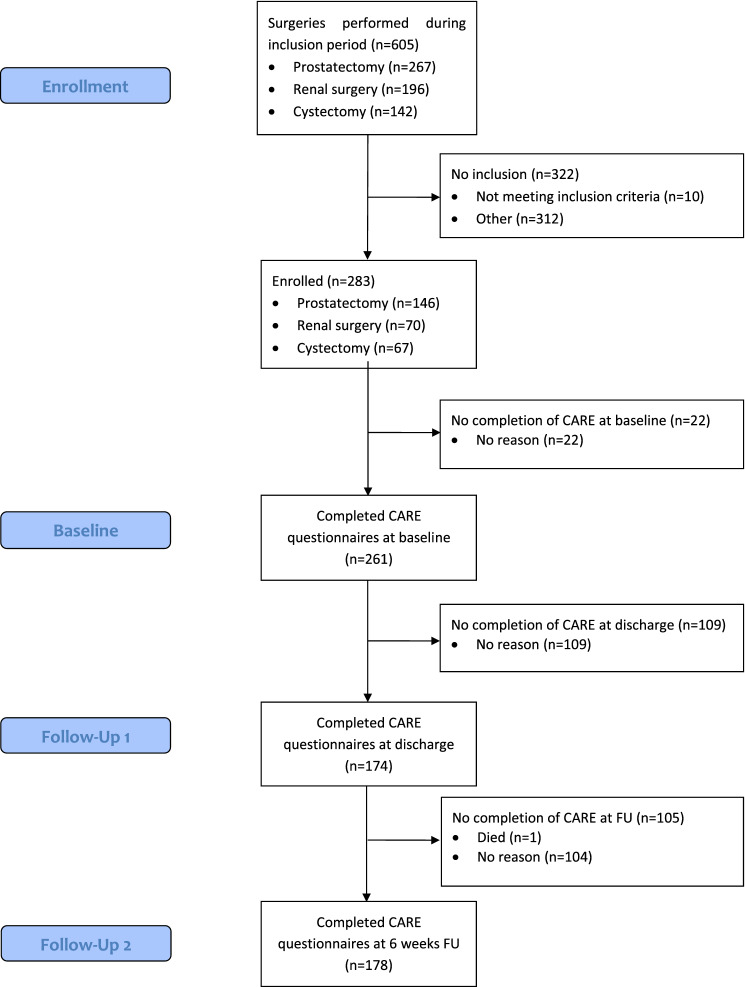
STROBE diagram

Overall, 283 patients were included in this study between April 2019 and August 2020 (RP 01/2020–08/2020, P/PN and RC 04/2019–08/2020), of which 146 underwent laparoscopic, robotic-assisted RP, 70 R/PN (35 open partial nephrectomy, 13 open radical nephrectomy, 20 robot-assisted partial nephrectomy, 2 robot-assisted radical nephrectomy) and 67 open RC, as depicted in Fig. 1. Of the 283 patients enrolled, 261 completed CARE at baseline, 174 completed it at discharge while 178 completed it 6 weeks postoperatively. One patient died during the follow-up phase.

### CARE results

**Fig. 2:**
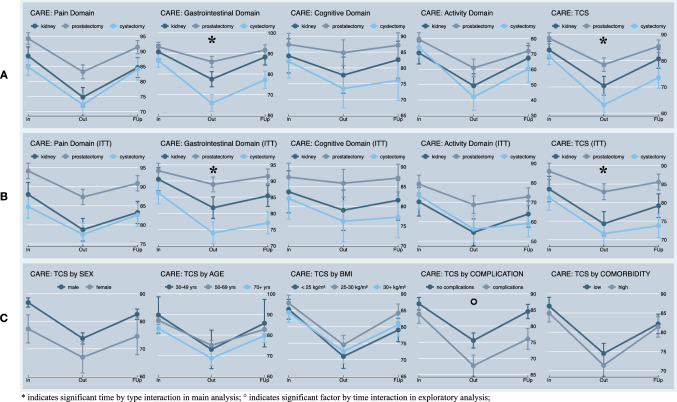
Mean results of CARE subscales over time and explanatory factor analysis. **a**, **b** Mean scores of the subscales of CARE at baseline (in), discharge (out) and follow-up (FUp) for the different types of operation, R/PN (kidney), RP (prostatectomy) and RC (cystectomy) based on the completer data (**a**) and intention to treat (ITT) data (**b**). **c** Exploratory analysis to identify the influence of sex, age, BMI, complication, and comorbidity on the total CARE score (TCS)

Detailed patient characteristics and oncological features are provided in Table 1 and the results of CARE in Table 2 in the appendix. In the baseline survey, all groups showed the lowest scores in the activity subscale (76.4 ± 30.6) while the highest scores were seen in the pain (90.7 ± 12.0) and gastrointestinal subscales (91.1 ± 11.9). At discharge, the activity subscale showed again the lowest scores for all groups (54.5 ± 24.8), while the highest scores were reported for the cognition subscale (81.7 ± 24.0). At follow-up, the order seen on the baseline survey was restored, with the highest reported scores for the pain (78.5 ± 14.1) and gastrointestinal subscales (78.9 ± 18.3) and the lowest scores for the activity subscale (69.0 ± 23.0). Figure 2 demonstrates the scores grouped by the type of surgery for the completer (Fig. 2a) and ITT data (Fig. 2b) and the TCS grouped by possible influencing factors (Fig. 2c).

**Table 1:** Patient characteristics

	All	Prostatectomy	Radical/partial nephrectomy	Cystectomy
Number of patients	283	146	70	67
Median age, IQR (years)	67 (61–72)	67.5 (62–71)	64 (55.5–72)	67 (61–75)
Male, *n* (%)	253 (89)	146 (100)	51 (73)	56 (84)
BMI, mean + std (kg/m^2^)^c^	27.82 ± 4.37	27.34 ± 3.57	29.02 ± 5.70	27.58 ± 4.14
Robotic surgery, *n* (%)	148 (50)	146 (100)	22 (31)	0 (0)
T0, T1 or Tis^b^, *n* (%)	60 (21)	0 (0)	42 (60)	18 (27)
T2, *n* (%)	116 (41)	100 (68)	3 (4)	13 (19)
T3, T4, *n* (%)	85 (30)	46 (32)	10 (14)	29 (43)
Benign/other, *n* (%)	22 (8)	–	15 (21)	7 (10)
Nodal positive N + , *n* (%)	26 (12)	11 (8)	1 (5)	14 (24)
Gleason score > 7a^a^	–	46 (31)	–	–
Continent UD, *n* (%)^b^	–	–	–	33 (50)
Median postoperative length of stay, IQR (days)	6 (6–11)	6 (6–6)	6 (5–7)	16 (12–22)
Complications, *n* (%)	68 (24%)	10 (7)	16 (23)	42 (63)
II, *n* (%) IIIa, *n* (%) IIIb, *n* (%) IVa, *n* (%) IVb, *n *(%) V, *n *(%)	47 (18) 6 (2) 9 (3) 3 (1) 2 (7) 1 (0.3)	4 (3) 2 (1) 1 (1) 2 (1) 1 (1) 0	12 (17) 2 (3) 2 (3) 0 0 0	31 (46) 2 (3) 6 (9) 1 (1) 1 (1) 1 (1)

^a^Prostate cancer only

^b^Bladder cancer only

^c^4 values missing

### Internal consistency and confirmatory factor analyses of the German translation of CARE

**Fig. 3:**
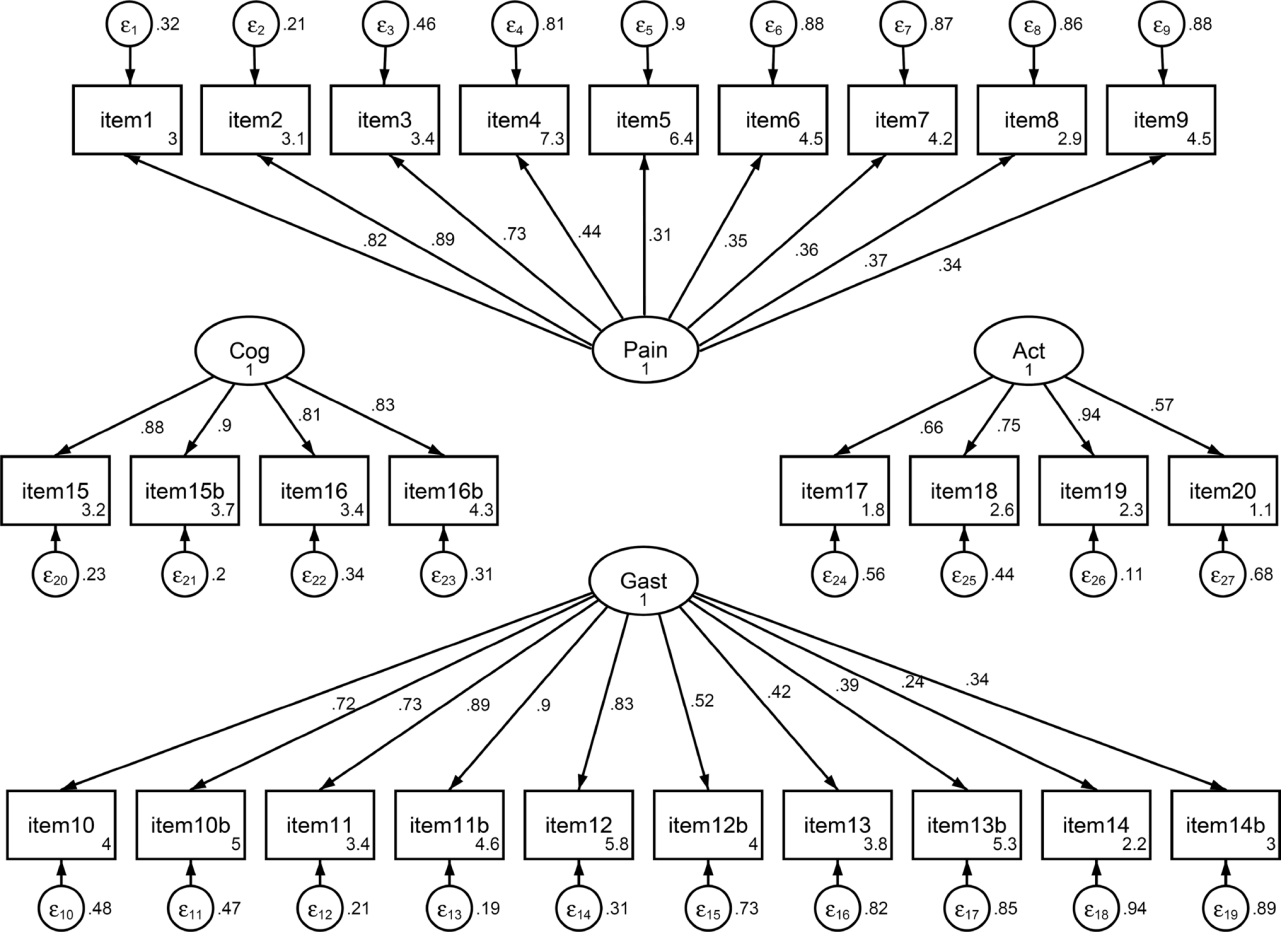
Results from confirmatory factor analyses; illustrated are the four CARE subscales and their respective item loadings. ε: standardized variance of the respective item; numbers in square items boxes refer to the constant of the standardized intercept; numbers next to arrows illustrate the standardized beta coefficient of the factor loading on the respective scale

**Fig. 4:**
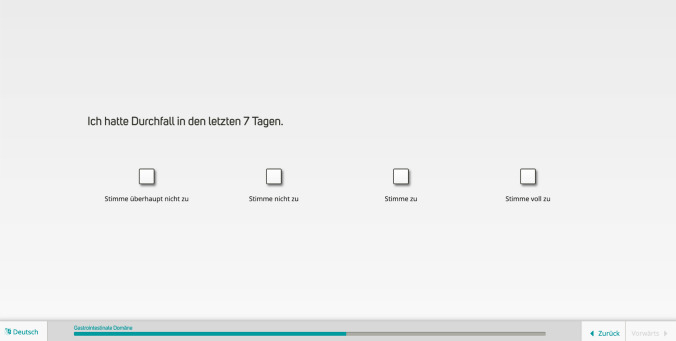
Screenshot of the heartbeat ePROM system; this image shows the German translation of the item “I have had diarrhea over the past seven days” as it is displayed for the patient in the heartbeat ePROM system. The answer that suits the patient can be selected simply by clicking on the box. At the bottom right, it is possible to go back to the previous questions. At the bottom center, the current progress and the current domain (subscale) are shown

Based on all the available data (all time-points), we found a high internal consistency in the respective subscales, namely pain (α = 0.775); gastrointestinal (α = 0.854); cognition (α = 0.920); and activity (α = 0.794). The lowest internal consistency was found in the TCS (α = 0.649). The inter-scale correlations of the subscales showed overall low correlations *r* < 0.6 confirming the measure of unique constructs with high correlation between TCS and the subscales, as depicted in Table 3 in the Appendix. The CFA revealed good factor loading for all items of the cognition and activity subscale as demonstrated in Fig. 3. However, in the pain and gastrointestinal subscales, in 6/9 and 4/10 items a factor loading < 0.5 was found.

### Main analyses

The main analyses on the completer data nested in each individual patient, investigating the time of the survey, type of surgery as well as their interaction as predictors, showed significant model fit for the CARE subscales of pain ($\chi_{(8)}^{2}$ = 237.45, *p* < *0.0001*), gastrointestinal ($\chi_{(8)}^{2}$ = 217.94, *p* < *0.0001*), cognition ($\chi_{(8)}^{2}$ = 25.98, *p* = *0.001*), activity ($\chi_{(8)}^{2}$ = 111.00, *p* < *0.0001*) and the TCS ($\chi_{(8)}^{2}$ = 234.17, *p* < *0.0001*)—indicating that both predictors explained significant variance in the outcome of interest. All models showed significant main effects of time of the survey, independent of the type of surgery, as depicted in Table 4 in the appendix, indicating a significant initial decline in HS from admission to discharge followed by a significant increase towards follow-up. In turn, all models also showed significant main effects of type of surgery, independent of time, indicating the greatest HS in patients undergoing RP, followed by R/PN, and the lowest with RC. All main effects of type of surgery remained after adjusting for sex, to account for the fact that prostatectomy was only conducted in male patients. Significant time by type interactions were observed on the subscale of gastrointestinal ($\chi_{(4)}^{2}$ = 30.37, *p* < *0.0001*) and the TCS ($\chi_{(4)}^{2}$ = 13.47, *p* = *0.009*). As illustrated in Fig. 2a, patients who underwent RC showed a significantly greater initial decline and worse recovery at follow-up on the respective subscale and the TCS. Again, the later interactions remained after adjusting for sex.

The ITT analyses following the imputation of missing data largely replicated the findings from the analyses on the completer data. Thus, we can conclude that the observed effects remain significant even when accounting for missing data and drop-out.

### Exploratory analyses

Exploratory analyses on the completer data showed a significant main effect of sex ($\chi_{(2)}^{2}$ = 11.94, *p* = *0.0001*) but no significant sex by time interaction ($\chi_{(4)}^{2}$ = 0.76, *p* = *0.68*). The findings illustrate better HS in males in this sample, independent of time. The effect was stable when adjusting for type of surgery ($\chi_{(1)}^{2}$ = 15.80, *p* = *0.0001*), to account for the fact that prostatectomy was only conducted in male patients.

Age (continuous) showed no significant main effects or interaction with time. Similarly, BMI (continuous) showed no effects. Significant main ($\chi_{(2)}^{2}$ = 18.36, *p* < *0.0001*) and interaction effects with time ($\chi_{(2)}^{2}$ = 8.61, *p* = *0.014*) emerged for complications. As illustrated in Fig. 2c, for patients in whom complications were recorded, the decline in HS from admission to discharge was significantly steeper and they did not achieve a comparable level of recovery at follow-up. Main effects on HS were observed for comorbidity (continuous, *p* = *0.011*). However, trajectories over time did not significantly differ as a function of comorbidity (continuous) or for groups based on comorbidity.

### Correlation of CARE with QLQ-C30

The TCS at discharge significantly correlated with global health (*r*_(57)_ = 0.509, *p* = *0.0001*) and physical function scores (*r*_(57)_ = 0.522, *p* < *0.0001*) in patients undergoing RC and R/PN. As illustrated in Appendix Fig. 5, greater HS at discharge was associated with better QLQ-C30 outcomes at follow-up. In a multivariable analysis, a TCS > 70 at discharge proved to be the only significant predictor for QLQ-C30 global health > 70 at follow-up (odds ratio = 5.26, 95%-confidence interval 1.42–19.37, *p* = *0.013*), as illustrated in Table 5 in the Appendix.

## Discussion

In this study, we assessed patient-reported postoperative convalescence after major urological surgery using the CARE questionnaire. We were able to demonstrate that the translated questionnaire was a valid ePROM to investigate postoperative recovery after major urological surgery. It was shown that the type of surgery and the occurrence of complications significantly influenced recovery.

This study was part of a larger project with the aim of digitally recording patient-reported outcomes after urological surgery. For this purpose, we introduced the ePROM system as described. The potential to improve patient care by using ePROMs has already been demonstrated from both the patient and clinician perspective, as it can simplify the process of sending out, answering, collecting and evaluating the questionnaires [20]. In addition, patient-centered care can be significantly improved through better identification of individual problem areas in cancer patients undergoing systemic treatment [21]. These advantages are in line with our experience. One unique feature of ePROMs is the possibility to monitor and, therefore, better react to side effects of therapies in real time, by, for example, using electronic symptom monitor systems [22]. While, in our study in the current phase, the focus has not yet been on monitoring, this is planned for the future, and it is quite conceivable, judging by the results up to this point. The ePROMS can, however, also have disadvantages, such as the exclusion of patients who are not comfortable using PCs/smartphones and the fact that severely ill patients often struggle to complete ePROMs [20]. In our study, we observed relatively high dropout and low inclusion rates, especially at the beginning of the trial when the ePROM system was newly introduced, which can be explained by the mentioned disadvantages, among other things. In addition, patients received multiple questionnaires in addition to the CARE, especially in the preoperative survey, which meant that some patients did not want to participate due to the size of the survey.

Several studies have shown the need for a current evaluation of post-discharge state of recovery [11, 23]. We chose the CARE questionnaire as the main ePROM for this study, as it has been proven to accurately report on postoperative convalescence and rehabilitation, is independent of the type of surgery and underlying conditions and is multidimensional [12, 13]. Since only German patients were included in the study, we translated the questionnaire into German. While the initial validation study reported a high internal consistency with Cronbach’s α > 0.70 for all of the assessment periods [12], in this study, we found even greater internal consistency of the respective subscales, using data from all available assessments. In line with Hollenbeck et al., we found similar inter correlations between CARE composite (TCS) and domain (subscales) scores by the time of assessment [12]. Correlations between subscales and the TCS in our study were slightly lower (0.660–0.748) than reported by Hollenbeck et al. (0.70–0.81). In line with Hollenbeck et al., we found lowest correlations between the CARE activity and CARE pain as well as CARE gastrointestinal subscale, underlining that each subscale measures separate variables. Furthermore, we used SEM to evaluate the CFA of the scale. In the absence of a detailed reporting of the initial factor analysis of the original scale, that would enable comparisons, we can only derive recommendations based on the current assessment in the present sample. In principle, factor-loadings of single items for the CARE cognitive and activity subscale were satisfactory. Both subscales showed superior performance against the pain and gastrointestinal subscales as indicated by the better model fit. Model fit for the later subscales may be improved by eliminating item with lower factor loadings (e.g., item 14 or item 5). However, here we aimed to present a first German translation of the scale and report on its utility in clinical practice. The detailed reporting of our CFA may guide further use and refinement of the scale in future studies.

The analysis of the reported scores of CARE showed, as expected, that time of survey significantly influenced CARE results. There was a significant decrease for all reported subscales and the TCS at the time of discharge with a significant increase 6 weeks postoperatively. These findings show that major urological surgery has a significant impact on the patients’ HS but, generally, good recovery is achieved 6 weeks after surgery. This finding is concordant to a study by Von Mechow et al. that showed that, after radical prostatectomy, patients were, on average, able to return to work by postoperative day 42 [24]. Considering the specific subscales, the highest decrease was found in the activity subscale at discharge and follow-up. It has been shown that better physical status and activity is associated with higher QoL [10]. Therefore, patients should be motivated to be moderately active early, soon after surgery. In contrast to the activity subscale, the pain subscale showed only moderate decreases at discharge and significant improvements at follow-up.

Type of surgery showed significant main effects, with the highest scores reported for the RP group, followed by the R/PN group and the worst scores for the RC group, which concurs with other studies on short-term recovery and QoL after such surgeries [25, 26]. We further analyzed the type of surgery by time of survey interaction. The RC group showed significantly higher decreases in the gastrointestinal subscale and TCS only at time of discharge and a significantly lower level of recovery at follow-up. While the morbidity of this operation is well documented [27, 28], this again emphasizes that cystectomy patients suffer remarkably from the operation. Our findings are concordant with a study by Stegemann et al. in 2012 that showed that patients achieved at least 90% of the baseline scores by the 90-day follow-up for all subscales, except the gastrointestinal subscale after robotic-assisted RC [14]. Other studies with longer follow-ups have shown that complete recovery after RC might take up to six months after open or robotic-assisted RC [29, 30]. Thus, outpatient care of RC patients should focus on the impairment in gastrointestinal function.

In the explanatory analysis on possible influencing factors, only the occurrence of a complication showed a significant effect on the TCS, with a greater decrease seen in patients experiencing a complication. Although discharge is usually delayed for these patients, they do not seem to be at the same level of recovery at discharge and follow-up and, thus, need a more intensive outpatient care.

Finally, we assessed whether the TCS can predict better HRQoL. We found a significant correlation of the TCS at discharge with QLQ-C30 outcomes at 8 weeks follow-up. This finding was confirmed in a multivariable analysis that shows that the state of recovery after surgery significantly influences QoL in cancer patients.

### Limitations

First, it has to be mentioned that different types of surgeries were included, thus, leading to a heterogenous overall study population. However, one aim of this study was to assess postoperative recovery among surgeries of different severity and impact with the CARE questionnaire, which inevitably led to this heterogeneity. Second, due to the exploratory nature of this study, no power analysis was performed. Third, we noticed a low inclusion rate and a high dropout rate due to the previously mentioned reasons. On this point, the lack of evaluation of the user experience could also be considered as a shortcoming. Fourth, this was a single center trial, and the results should be confirmed in a multicenter trial. Due to these limitations, the study results must be interpreted with caution.

## Conclusion

This German translation of the CARE questionnaire proved to be a valid ePROM to assess patient-reported postoperative recovery after major urological surgery and to predict QoL after surgery. Subsequent studies could investigate the extent to which the use of CARE as part of criteria for discharge can improve quality of life after discharge and correlates to early readmissions.

## Figures and Tables

**Fig. 5:**
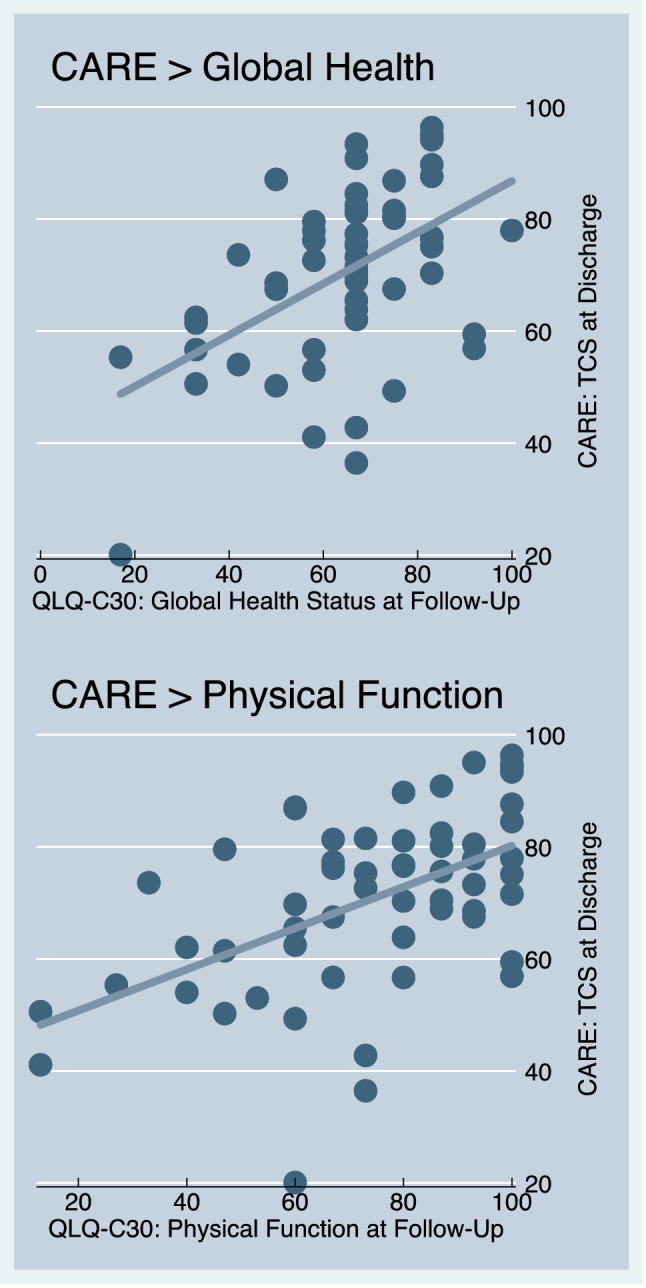
Pearson’s correlation of TCS with QLQ-C30 (cystectomy and renal surgery only). Top: correlation TCS (discharge) and QLQ-C30 global health (follow-up). Bottom: correlation TCS (discharge) and QLQ-C30 physical function (follow-up)

## Appendix

See Tables 2, 3, 4 and 5 and Figs. 4 and 5.

**Table 2:** Absolute scores of CARE questionnaire (completer data)

	TCS	Pain	Gastrointestinal	Cognition	Activity
Baseline^a^					
All	86.0 ± 12.7	90.7 ± 12.0	91.1 ± 11.9	85.5 ± 19.9	76.4 ± 30.6
RP	88.6 ± 10.1	94.3 ± 7.7	93.2 ± 8.5	87.4 ± 16.6	79.3 ± 30.7
R/PN	84.3 ± 14.2	88.4 ± 13.4	90.8 ± 12.8	84.4 ± 24.3	70.8 ± 33.5
RC	81.4 ± 15.4	84.7 ± 15.4	86.5 ± 15.9	82.3 ± 22.0	74.2 ± 27.0
Discharge^b^					
All	73.5 ± 15.0	78.5 ± 14.1	78.9 ± 18.3	81.7 ± 24.0	54.5 ± 24.8
RP	78.3 ± 12.0	83.2 ± 10.9	85.8 ± 13.6	84.5 ± 23.4	61.0 ± 21.5
R/PN	71.3 ± 14.4	75.1 ± 19.5	77.2 ± 19.5	79.2 ± 26.0	50.3 ± 23.8
RC	64.6 ± 17.8	71.9 ± 16.7	65.1 ± 18.5	78.3 ± 22.3	44.0 ± 29.3
6 weeks FU^c^					
All	82.5 ± 12.6	88.7 ± 11.2	88.5 ± 13.2	84.3 ± 21.1	69.0 ± 23.0
RP	85.6 ± 9.9	91.9 ± 8.8	91.6 ± 9.7	86.9 ± 18.5	71.7 ± 22.6
R/PN	81.0 ± 13.6	85.1 ± 13.1	88.7 ± 11.8	83.8 ± 22.6	67.8 ± 22.6
RC	75.3 ± 15.4	84.2 ± 12.3	79.2 ± 18.3	77.7 ± 24.8	62.7 ± 23.9

Mean ± standard deviation was used for all reported scores

^a^*n* = 22 not completed questionnaires

^b^*n* = 109 not completed questionnairs

^c^*n* = 105 not completed questionnairs

**Table 3:** Pearson’s correlation coefficients between CARE composite (total) and domain (subscales) scores for all time-points

	Baseline					Discharge					Follow-up				
	Total	Pain	Gast	Cog	Activ	Total	Pain	Gast	Cog	Activ	Total	Pain	Gast	Cog	Activ
Total (*r*)	1					1					1				
*p-*value	< *0.0001*					< *0.0001*					< *0.0001*				
Pain (*r*)	0.660	1				0.730	1				0.726	1			
*p-*value	< *0.0001*	< *0.0001*				< *0.0001*	< *0.0001*				< *0.0001*	< *0.0001*			
Gastrointestinal (*r*)	0.666	0.635	1			0.720	0.464	1			0.738	0.609	1		
*p-*value	< *0.0001*	< *0.0001*	< *0.0001*			< *0.0001*	< *0.0001*	< *0.0001*			< *0.0001*	< *0.0001*	< *0.0001*		
Cognitive (*r*)	0.674	0.425	0.572	1		0.744	0.453	0.418	1		0.740	0.417	0.449	1	
*p-*value	< *0.0001*	< *0.0001*	< *0.0001*	< *0.0001*		< *0.0001*	< *0.0001*	< *0.0001*	< *0.0001*		< *0.0001*	< *0.0001*	< *0.0001*	< *0.0001*	
Activity (*r*)	0.714	0.196	0.114	.0103	1	0.748	0.460	0.379	0.278	1	0.717	0.354	0.321	0.227	1
*p-*value	< *0.0001*	*0.002*	*0.071*	*0.101*	< *0.0001*	< *0.0001*	< *0.0001*	< *0.0001*	*0.001*	< *0.0001*	< *0.0001*	< *0.0001*	< *0.0001*	*0.003*	< *0.0001*

**Table 4:** Main effects of time of survey and type of surgery (completer data)

	TCS		Pain		Gastrointestinal		Cognition		Activity	
Time of survey (baseline, discharge, follow-up)										
$\chi_{(2)}^{2}$		183.68		170.19		159.25		15.29		89.13
*p*-value		< *0.0001*		< *0.0001*		< *0.0001*		*0.0005*		< *0.0001*
Type of surgery (RP, R/PN, RC)										
$\chi_{(2)}^{2}$		46.33		47.10		62.45		10.46		14.70
*p*-value		< 0.0001		< 0.0001		< 0.0001		0.005		0.0006
Time by type interaction										
$\chi_{(4)}^{2}$		13.47		3.16		30.37		4.67		6.63
*p*-value		*0.009*		*0.53*		< *0.0001*		*0.32*		*0.16*

Mixed-linear regression models were used for all calculations of Chi^2^(χ^2^) and respective *p*-values

**Table 5:** Multivariable logistic regression analysis for the prediction of QLQ-C30 global health score > 70 at 8 weeks follow-up (*n* = 81)

	Wald χ^2^	Odds ratio	95% Confidence interval	*p*-value
TCS discharge > 70 (yes vs. no)	6.19	5.26	1.42–19.47	**0.013**
Type of surgery (renal surgery vs. cystectomy)	0.59	1.71	0.44–6.68	0.442
Sex (male vs. female)	0.46	1.75	0.34–8.83	0.498
BMI > 30 (yes vs. no)	0.28	0.67	0.17–2.74	0.594
CCI > 2 (yes vs. no)	0.16	1.3	0.34–4.34	0.688
Complication (yes vs. no)	0.09	1.22	0.34–4.34	0.760

Model fit: χ^2^ = 8.23, *p* = 0.2; model fit after removal of BMI, CCI and complication: 8.34, *p* = 0.039

## Footnotes

1 heartbeat ONE by Heartbeat medical Solution GmbH, Berlin. https://heartbeat-med.com
